# Climate warming restructures seasonal dynamics of grassland soil microbial communities

**DOI:** 10.1002/mlf2.12035

**Published:** 2022-09-15

**Authors:** Xue Guo, Mengting Yuan, Jiesi Lei, Zhou Shi, Xishu Zhou, Jiabao Li, Ye Deng, Yunfeng Yang, Liyou Wu, Yiqi Luo, James M. Tiedje, Jizhong Zhou

**Affiliations:** ^1^ State Key Joint Laboratory of Environment Simulation and Pollution Control, School of Environment Tsinghua University Beijing China; ^2^ Institute for Environmental Genomics and Department of Microbiology and Plant Biology University of Oklahoma Norman Oklahoma USA; ^3^ Department of Environmental Science, Policy, and Management University of California Berkeley California USA; ^4^ Gladstone Institutes and Chan‐Zuckerberg Biohub San Francisco California USA; ^5^ School of Minerals Processing and Bioengineering Central South University Changsha China; ^6^ Key Laboratory of Environmental and Applied Microbiology, Chinese Academy of Sciences and Environmental Microbiology, & Key Laboratory of Sichuan Province, Chengdu Institute of Biology Chinese Academy of Sciences Chengdu China; ^7^ Key Laboratory of Environmental Biotechnology, Research Center for Eco‐Environmental Sciences Chinese Academy of Sciences Beijing China; ^8^ Department of Biological Sciences, Center for Ecosystem Science and Society Northern Arizona University Flagstaff Arizona USA; ^9^ Center for Microbial Ecology Michigan State University East Lansing Michigan USA; ^10^ School of Civil Engineering and Environmental Sciences University of Oklahoma Norman Oklahoma USA; ^11^ Earth and Environmental Sciences Lawrence Berkeley National Laboratory Berkeley California USA; ^12^ School of Computer Sciences University of Oklahoma Norman Oklahoma USA

**Keywords:** climate warming, microbial community, microbial network, seasonal dynamics

## Abstract

Soil microbial community's responses to climate warming alter the global carbon cycle. In temperate ecosystems, soil microbial communities function along seasonal cycles. However, little is known about how the responses of soil microbial communities to warming vary when the season changes. In this study, we investigated the seasonal dynamics of soil bacterial community under experimental warming in a temperate tall‐grass prairie ecosystem. Our results showed that warming significantly (*p* = 0.001) shifted community structure, such that the differences of microbial communities between warming and control plots increased nonlinearly (*R* ^2^ = 0.578, *p* = 0.021) from spring to winter. Also, warming significantly (*p* < 0.050) increased microbial network complexity and robustness, especially during the colder seasons, despite large variations in network size and complexity in different seasons. In addition, the relative importance of stochastic processes in shaping the microbial community decreased by warming in fall and winter but not in spring and summer. Our study indicates that climate warming restructures the seasonal dynamics of soil microbial community in a temperate ecosystem. Such seasonality of microbial responses to warming may enlarge over time and could have significant impacts on the terrestrial carbon cycle.

## Impact statement

The feedback response of soil microbial communities to climate warming is one of the major uncertainties in projecting future climate scenarios. Despite intensive studies for two decades, such feedback and associated mechanisms are still poorly understood with regard to seasonal variation. In this study, we revealed that warming treatments restructured seasonal dynamics of the microbial community, resulting in larger differences in the community structure and network patterns in winter months than in warmer months. This study suggests that future climate changes in seasonality might shift soil microbial community phenology and associated functioning. Such information about the seasonal dynamics of soil microbial communities is valuable for predicting the long‐term global carbon cycle.

## INTRODUCTION

The acceleration of climate warming has become a scientific, political, and economic concern^1^. Climate warming disturbs carbon (C) and nitrogen cycling between Earth's atmosphere and terrestrial ecosystems, but it is not clear whether the emission of greenhouse gases (i.e., CO_2_, CH_4_, and N_2_O) from soils will further increase via warming‐induced positive feedback^2^, ^3^. The shifts of soil greenhouse gas emission under climate warming substantially depend on microbial communities, since they are critical mediators of the terrestrial C cycle by playing a major role in C mineralization and stabilization^4^, ^5^, and affecting soil health and nutrient availability to plants^6^. Therefore, how soil microbial communities and their ecosystem functions are influenced by climate warming is critical for projecting future climatic regimes.

Most studies on ecosystem responses to climate warming only discussed warming effects with yearly based observations^7^, ^8^, ^9^, despite strong seasonal variations in ecological community diversity and structure^10^, ^11^. Seasonal dynamics of ecological communities is critical for evaluating ecosystem functions and services, and their responses to environmental changes in the long term^12^, ^13^. For example, shifts in plant and animal phenology, such as flowering time and migration pattern, are among the most sensitive signs of climate change^14^, ^15^. A wide range of environmental factors that affect soil microbial responses to climate warming undergo seasonal cycles in temperate ecosystems^12^, ^16^, ^17^, such as precipitation^18^, moisture^19^, resource availability^20^, and the intensity of disturbances like clipping or grazing^21^. Thus, microbial responses to climate warming should be considered with regard to seasonal variations^10^, ^11^, ^13^, ^22^, ^23^. With the decrease in sequencing cost and increase in our capacity to analyze environmental microbial samples, the temporal dynamics of soil microbial communities and their functions can be captured in a higher resolution for a better prediction of the terrestrial C cycle.

Relationships among taxa embody a dimension of information beyond species diversity and composition in community assemblage^24^ and are reflected in covariation of species abundances^25^. How microbes assemble and covary in soil may signify their responses to edaphic factors, niche sharing and partition, interspecies interactions, and various stochastic processes. The mechanisms by which soil microbes assemble and the pattern by which they covary are strongly influenced by plant growth^26^ and resource availability^27^, which show strong seasonal changes. However, few reports are available on how soil microbial communities assemble and covary in different seasons. In our yearly based long‐term observations, deterministic processes became stronger in shaping soil microbial communities under warming, possibly due to environmental filtering by warming‐related changes in soil abiotic conditions^8^. Microbial networks became more complex and resistant over a few years with prolonged warming^9^. It is unclear whether such community organizational shifts also happen with shorter‐term but stronger seasonal temperature variation, and with different growth stages and composition of plants, especially in conjunction with climate warming.

To understand whether and how the effects of climate warming on soil microbial communities differ along with the season, we examined month‐to‐month changes in soil microbial communities under experimental warming in a temperate tall‐grass prairie ecosystem of the US Great Plains in Central Oklahoma (34̊°59ʹN, 97̊°31ʹW). We aimed to determine: (i) whether and how experimental warming alters the soil microbial community structure during different seasons; (ii) how experimental warming impacts seasonal dynamics of soil microbial network patterns; (iii) whether and how warming‐induced changes in microbial community structure and network patterns shape ecosystem functioning (i.e., ecosystem C fluxes and soil respirations). Our results revealed that climate warming strongly affected the seasonal succession of microbial community structure and network patterns, which significantly contribute to ecosystem functioning.

## RESULTS

### Seasonality of soil variables, soil respirations, and ecosystem C fluxes

**Figure 1:**
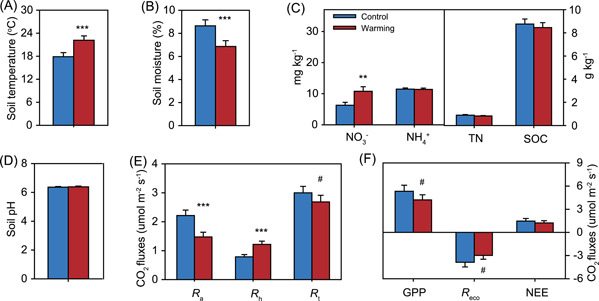
Warming effects on soil variables, soil respirations, and ecosystem carbon fluxes. (A−D) Soil surface (0−15 cm) temperature, moisture, NO_3_^−^, NH_4_^+^, TN, SOC, and pH, averaged from four replicated plots across the entire year. (E) In situ soil respirations measured in the field plots. (F) Gross primary productivity (GPP), ecosystem respiration (*R*_eco_), and net ecosystem exchange (NEE). Positive values indicate carbon sink, and negative values represent carbon source. Blue and red colors denote control and warming treatments, respectively. Error bars represent standard errors. The differences between warming and control conditions were tested by linear mixed‐effects models, indicated by *** when *p* < 0.001, ** when *p* < 0.01, # when *p* < 0.10. More information is shown in Figure S1 and Table S1. *R*_a_, autotrophic respiration derived from plant roots; *R*_h_, heterotrophic respiration from soil microbes, and *R*_t_, soil total respiration; SOC, soil organic carbon; TN, total nitrogen.

Soil variables and ecosystem C fluxes exhibited strong seasonal fluctuations and were impacted by the warming treatment (Figures 1A−F and S1A−M and Table S1). Soil temperature, moisture, soil organic carbon (SOC), nitrate (NO_3_^−^), ammonia (NH_4_^+^), pH, soil total respiration (*R*_t_), heterotrophic respiration (*R*_h_), autotrophic/root respiration (*R*_a_), and ecosystem C fluxes, including ecosystem respiration (*R*_eco_), gross primary productivity (GPP), and net ecosystem exchange (NEE) varied over time (*p* < 0.02). Warming increased (*p* < 0.001) soil temperature by 4.3°C and decreased (*p* < 0.001) soil moisture by 20.7% averaged for the year (Figure 1A,B). The impacts of warming on soil temperature and moisture were consistent over time (*p* > 0.05 for the warming × month interaction; Table S1). Soil nitrate contents were higher (*p* = 0.001) in warmed plots than in control plots, with an increase of 71.2% across the entire year (Figure 1C). Warming increased *R*_h_ by 54.4% (*p* < 0.001), but decreased *R*_a_ by 33.4% (*p* < 0.001). Consequently, *R*_t_ was not affected by warming across the entire year (Figure 1E). In addition, significant variations in the warming effect on *R*_h_ were observed over time (*p* = 0.033 for the warming × month interaction; Table S1). Although no significant warming effects were observed on *R*_eco_, GPP, and NEE over the year (Figure 1F), warming decreased both *R*_eco_ and GPP during the months of plant biomass peak (March and September) more than in the other months (Figure S1 and Table S1).

### Seasonal changes in microbial community structure

**Figure 2:**
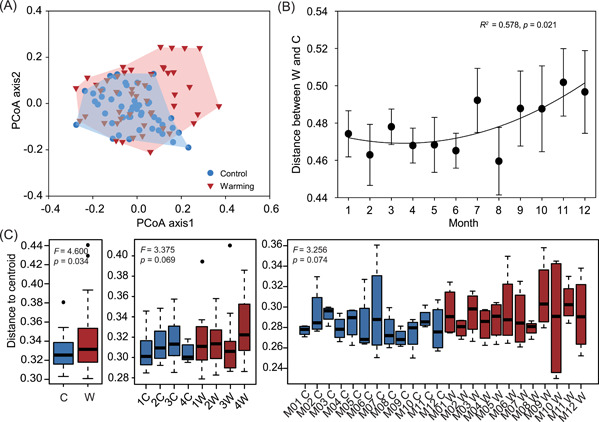
Microbial community beta‐diversity indicated by the multivariate homogeneity of group variances. (A) PCoA of soil bacterial community structure under warming and control treatments for 12 months, based on the Bray−Curtis distance metrics. (B) Community distances of bacterial community between warming (W) and control (C) conditions over time. The temporal change of dissimilarity between warmed and control plots in each block was fitted to nonlinear quadratic regression. The *R* ^2^ reflects the variance explained by the regression. (C) The distance of samples to group centroids by treatment, experimental plot (distance to centroid represents the temporal variation of communities), and treatment × sampling time (distance to centroid represents a spatial variation of the four field blocks). Distances to group centroids are significantly larger for warmed than control communities when grouping by treatment (*F* = 4.600, *p* = 0.022), experimental plots (*F* = 3.375, *p* = 0.069), or treatment × sampling time (*F* = 3.256, *p* = 0.074). The labels of 1C−4C represent four control replicates, and 1W−4W represent four warmed replicates, respectively. M01 C−M12 C represent control communities in 12 different months, and M01 W−M12 W represent warmed communities in 12 different months, respectively. PCoA, principal coordinate analysis.

The microbial communities under warming tended to deviate from those of the control, as indicated by principal coordinate analysis (PCoA) based on the Bray−Curtis distance metrics (Figure 2A). The main effects of sampling month and warming significantly (*F* , 1.856−3.792; *p* < 0.001) affected soil microbial community structure, explaining 18.1% and 3.4% of the total variation, respectively (Table 1), and warming differentially affected taxa over time (Figure S2). Within each month, warming significantly (*p* < 0.05) increased the relative abundances of *Firmicutes* in January, *Actinobacteria* in April, and *Gemmatimonadetes* in May (Figure S2). Decreased relative abundances under warming were observed for *Deltaproteobacteria* in February, *Betaproteobacteria* in April, *Deltaproteobacteria* in October, and *Planctomycetes* in November (Figure S2). Across all months, warming significantly (*p* < 0.05) increased the relative abundances of *Actinobacteria*, *Firmicutes*, and *Thaumarchaeota*, but decreased the relative abundances of *Deltaproteobacteria* and *Planctomycetes* (Figure S2).

**Table 1:** The effects of warming and month on microbial community structures tested by permutational multivariate analysis of variance.

	*F*	*R* ^2^	*p*
Warming	3.792	0.034	**0.001**
Month	1.856	0.181	**0.001**
Warming × month	0.996	0.098	0.447

Permutational multivariate analysis of variance (PERMANOVA) was performed with the Bray−Curtis dissimilarity metric. The two‐way repeated‐measures ANOVA model was set as “dissimilarity~warming × month + block” using function adonis in R package vegan. The degree of freedom was 1 for warming treatment, 11 for month, and 69 for residuals. Significant effects (*p* ≤ 0.05) are shown in bold.

The difference in microbial community structure between warming and control treatments increased nonlinearly (*R* ^2^ = 0.578, *p* = 0.021) from spring to winter (Figure 2B). Furthermore, the communities under warming exhibited larger dispersion than those under control (*F* = 4.600, *p* = 0.034; Figure 2C), consistent with the less closely clustered samples under warming in PCoA. Such larger community dispersion under warming was likely due to the increase in both temporal variation across sampling months and spatial heterogeneity among experimental plots (*p* < 0.074; Figure 2C).

### Seasonal dynamics of microbial networks

**Figure 3:**
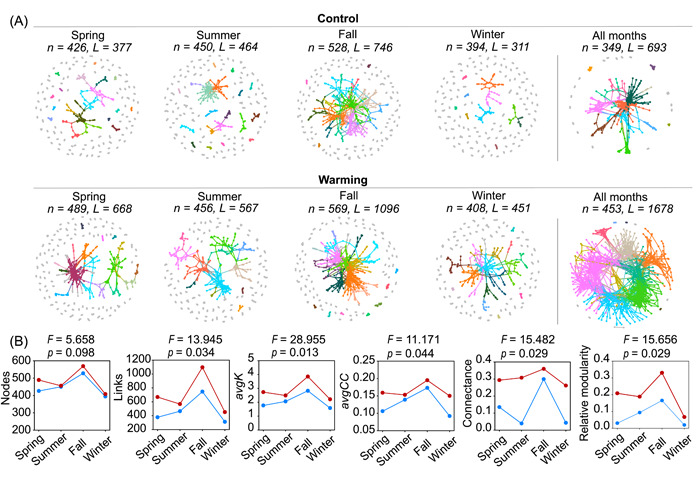
Soil microbial co‐occurrence networks from separated seasons and all time points. (A) Overall dynamics of microbial co‐occurrence networks under warming and control. Modules separated using Greedy algorithm are uniquely colored in each network for modules with >5 nodes, and in gray for modules with ≤5 nodes. Network size (*n*) and connectivity (*L*) are shown for each network. Details of network topological properties are listed in Table S2. (B) Seasonal change in network topological properties under warming (red) and control (blue), including network size (i.e., total number of nodes), connectivity (i.e., total number of links), average connectivity (*avgK*), average clustering coefficient (*avgCC*), connectance and relative modularity. Significance of warming effects on these topological properties was tested by linear mixed‐effects models with season as a random intercept effect. The *F* and *p* values are shown above each plot.

Eight seasonal networks and two global networks (see the “Materials and Methods” section) were constructed to explore the associations of microorganisms under warming and with seasons (Figure 3A). The seasonal networks were constructed with the correlation threshold *S*_t_ = 0.890. The network sizes (*n*) ranged from 394 to 569 nodes with 311−1096 links (Figure 3A). The global networks for warmed and control communities were constructed with a correlation threshold *S*_t_ = 0.680, such that networks had 453 and 349 nodes with 1678 and 693 links, respectively (Figure 3A and Table S2). Overall, species tended to co‐occur (83.1%−93.2% links from positive correlations) rather than co‐exclude (6.8%−16.9% links from negative correlations). All networks were scale‐free, as indicated by a good fit of the node degree distributions to power‐law functions (*R* ^2^, 0.850−0.977). They were also modular as shown by high modularity (*M* > 0.522) and exhibited small world behavior with geodesic distances (the average shortest path between paired nodes of 3.572−9.531; Table S2). These networks are differently structured from the random networks, indicating that the empirical networks are unlikely formed as such by random chance. All empirical networks have higher average clustering coefficient (*avgCC*) and modularity compared to their corresponding random networks, indicating that members in the empirical networks were driven to form clusters and nest in modules (Table S2).

Despite that the operational taxonomic units (OTUs) in warmed communities were on average 10.7% fewer than control communities, the seasonal and global networks under warming had 6.9% and 29.8% more nodes than the corresponding controls, respectively (Figure 3B and Table S2). This indicated that more species covaried in abundance under warming. Under warming, the seasonal networks had 46.6% more links on average (*F* = 13.945, *p* = 0.034), and the global network had 142.1% more links compared with the control, leading to a 36.9% increase (*F* = 28.955, *p* = 0.013) of average connectivity (*avgK*) (Figure 3B). Networks for warmed communities also had higher connectance, clustering coefficient (*avgCC*) and relative modularity^28^ than the control (*F* = 11.171−15.656, *p* < 0.044; Figure 3B).

The size, complexity, and taxonomic composition of nodes in major modules of soil microbial networks changed along with the season (Figures 3B and S3). Under both warming and control conditions, the smallest network was obtained in winter and the largest in fall. Compared to winter, the *avgK* was 12.1%−19.9%, 11.8%−30.6%, and 39.5%−78.9% higher in spring, summer, and fall, respectively. The differences in network size and complexity between warming and control were more profound in other seasons (3.6%−14.8% larger with 45%−77% more links under warming) than in summer (1.3% larger with 22.2% more links), consistent with the smallest difference of community structure in summer months (Figure 2B).

Module hubs, connectors, and network hubs were identified as key taxa in the network (Figure S4 and Table S3). One to six module hubs were identified in each of the seasonal networks. Thirty‐two of the 34 connectors occurred in fall and global networks. Warming networks have more key taxa than controls. Among all the seasons, fall networks had the most module hubs and connectors (Figure S4C). The number of connectors and module hubs coincided with the complexity of networks. The key taxa in both global and seasonal networks mostly belonged to abundant phyla, with 30% from *Actinobacteria* and 25% from *Acidobacteria* (Figures S4 and S5 and Table S3). However, some module hubs were low‐abundance, nondominant species, such as the *Gemmatimonadetes* hub in Module 1 of the fall‐warming network. Notably, some OTUs occurred as key taxa in more than one network (Table S3). For instance, OTU_21, belonging to the *Solirubrobacter* genus in *Actinobacteria*, was a module hub in fall‐control, winter‐control, and global‐control networks. Additional eight OTUs (e.g., three *Acidobacteria*, two *Actinobacteria*, one *Gammaproteobacteria*, one WPS‐1, and one unclassified phylum) served as module hubs or connectors in at least two networks.

**Figure 4:**
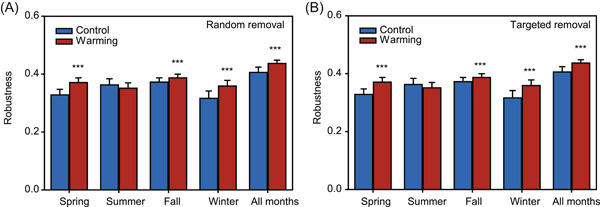
Stability of microbial networks measured by robustness. (A) Robustness represents the proportion of taxa that remained with 50% of the taxa randomly removed from each network. (B) Robustness represents the proportion of taxa that remained with five module hubs removed from each network. The error bar indicates the standard deviation of 100 repetitions of the simulation. Significant differences between warming and control were calculated by two‐sided *t*‐test and indicated by *** when *p* < 0.001.

The robustness of microbial networks, measured by the network's resistance to node loss, was evaluated by simulating random and targeted taxa extinction (Figure 4A,B). The global networks under warming had significantly (*p* < 0.001) higher robustness than those under control, based on either random taxa loss or targeted removal of module hubs (Figure 4A,B). Furthermore, warming significantly (*p* < 0.001) enhanced the robustness of microbial networks in all seasons except summer (Figure 4A,B).

### Microbial community assembly mechanisms

**Figure 5:**
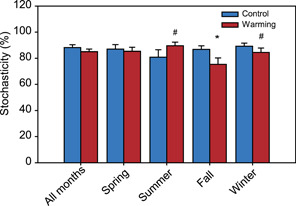
Community stochasticity under control and warming conditions. NST in control and warmed plots were calculated to quantify overall community stochasticity. The significant differences between warming and control conditions are indicated as *p < 0.05, and ^#^ *p* < 0.10 based on permutational multivariate analysis of variance (PERMANOVA). The error bars indicate standard errors. NST, normalized stochasticity ratio.

We calculated the normalized stochasticity ratio (NST) index to quantify the relative importance of stochastic and deterministic processes in microbial community assembly. Stochastic processes contributed to 75.3%−89.2% of the community variations under warming and control conditions (Figure 5), suggesting that stochasticity was the major process in determining microbial community structures. Across all months, warming exhibited no significant effects on stochasticity. Interestingly, warming decreased the relative importance of stochastic processes by 11.4% in fall and by 4.7% in winter, whereas warming increased the relative importance of stochastic processes by 8.7% in summer (*p* < 0.10).

### Links between microbial community and soil variables and ecosystem function

**Figure 6:**
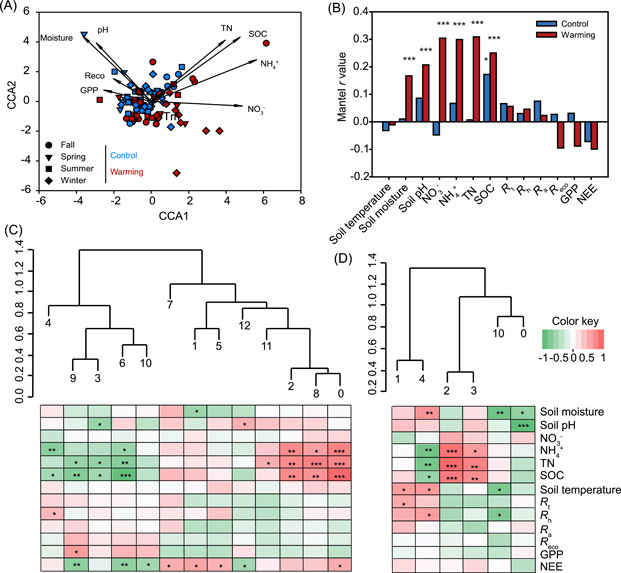
Microbial community structures link to soil variables and ecosystem C fluxes. (A) CCA of microbial communities and environmental variables, including GPP, *R*_eco_, soil moisture, pH, temperature (*T*_m_), SOC, TN, NO_3_^−^, and NH_4_^+^ contents. (B) Correlations between microbial community structures and environmental variables under warming and control treatments by Mantel tests. (C, D) Module eigengenes’ correlations with environmental variables for the control (C) and warmed (D) networks across all months. An eigengene was calculated for each module to represent all nodes within each module. Clusters show the hierarchical structure of eigengenes for modules numbered as in Figure S3. The Pearson correlation coefficient of each eigengene−environmental variable pair is indicated by the heatmap. Significances of these correlations are indicated by *** when *p* < 0.001, ** when *p* < 0.01, and * when *p* < 0.05. CCA, canonical correspondence analysis.

Canonical correspondence analysis (CCA) and Mantel tests were performed to assess the relationships of microbial community structure with soil properties, soil respirations, and ecosystem C fluxes (Figure 6A,B and Table S4). The CCA results indicated that microbial community structure was significantly (*F* = 1.435, *p* = 0.014) shaped by soil SOC, total nitrogen (TN), pH, moisture, and temperature (Figure 6A). Consistent with these results, Mantel tests revealed that seasonal succession of microbial community structure had stronger correlations with soil moisture, pH, SOC, and N species under warming than under control conditions (Figure 6B and Table S4).

To reveal the relationships among network modules, each module with more than five nodes was decomposed into a single representative abundance profile, the module eigengene. The module eigengenes explained 59%−83% variations of the module member abundance profiles in seasonal networks and 34%−69% in global networks (Table S5). The hierarchical clustering of the eigengenes was used to represent the similarities of modules. In the global control network, 13 modules were clustered into two major groups, whereas the 6 modules in the global warming network formed three distinct groups (Figure 6C,D). Modules in the seasonal networks under warming also formed different clusters compared with those in control networks (Figure S6).

The eigengene abundance profiles were correlated to the environmental profile to reveal the correlations of modules with environmental variables and ecosystem functioning (Table S5 and Figures 6C,D and S6). Generally, the modules in fall networks had more significant (19%−33% of total pairs of correlations with *p* < 0.05) correlations with edaphic conditions and ecosystem C fluxes, while the modules in winter and spring networks had less (5%−15% of total pairs of correlations with *p* < 0.05; Figure S6). The microbial substrate‐related variables (e.g., SOC, TN, and NH_4_^+^ contents) were significantly (*p* < 0.05) correlated to at least one module in all the networks, and to several modules in more complex networks. Modules in the global control network tended to correlate with GPP and NEE, but the modules in the global warming network had more correlations with soil temperature, moisture, *R*_h_, and *R*_t_ (Figure 6C,D).

## DISCUSSION

In temperate ecosystems, the weather and plant growth follow seasonal cycles.^29^ While climate warming is known to alter the phenology of plants and animals, to date, insufficient information is available on how climate warming affects the seasonal succession of microbial communities. Here, we report that despite large seasonal variations, warming changed the structure of soil microbial communities and increased the complexity of their networks in all the seasons, signifying a robust warming effect also observed over multiple years^8^, ^30^. Furthermore, warming altered the seasonal dynamics of microbial succession by imposing a stronger deterministic effect in winter than in the summer, leading to larger differences in community structure between warming and control plots. These effects may enlarge in the long term, resulting in considerable impacts on the overall soil C balance between land and atmosphere.

Consistent with the previous reports^31^, ^32^, ^33^, we also observed rapid changes in soil microbial community structure along seasons, with a larger seasonal effect than what was imposed by simulated warming. Microbial network size and complexity also changed along with the season, likely in response to changed soil microclimate and resource availability^34^, ^35^. From non‐growing season to growing season, soil temperature and moisture became favorable for more species. As most plants are annual in our field site, microbial substrates from plant exudates also increase along plant growth during this time frame^36^. With gradually increased microbial diversity and stronger environmental filtering from plant roots^26^, the networks became increasingly large and complex from non‐growing season to growing season.

Despite large seasonal variations, soil microbial communities responded to warming with altered taxonomic composition and increased network complexity in all seasons and across the entire year. Both microbial community structure and network modules are correlated with soil moisture, N availability, and SOC. The decrease in water content caused by warming could impede the movement of nutrients in soil^37^, leading to patches of communities that spatially co‐occur (e.g., dispersal limitation); it may also cause nutrient depletion and promote microbial cooperation and competition^25^, ^38^, ^39^. Meanwhile, warming could favor microorganisms for the decomposition of high C/N ratio substrates^40^, ^41^, which may restructure the networks.

Soil sampling on a monthly basis enabled us to capture uneven warming effects on microbial communities in different seasons. Warming effects on both community structure and microbial networks were stronger in the winter months and weaker in the summer months. We also found that in fall and winter, warming tended to increase the importance of deterministic processes (e.g., selection) in driving community assembly, which is consistent with our long‐term observations conducted every year in the fall^8^. In contrast, warmed communities showed higher stochasticity in summer, suggesting that selection imposed by warming was weaker. The magnitude of the warming effect on the community across seasons is consistent with the relative importance of selection imposed by warming, as opposed to stochasticity caused by warming. We did not see a larger warming effect on soil temperature in winter. However, soil moisture was consistently decreased by warming spanning the fall and winter months of October to December, January, and February, but was not changed by warming in the spring and summer months. It is possible that warming‐induced moisture decrease imposes deterministic selections on the community^37^ and then leads to larger differences in microbial communities between warming and control conditions in the winter months.

Notably, warming tended to increase both the temporal and spatial variations of soil microbial community, possibly implying faster species turnover in warmer conditions. This mirrors our long‐term observation that warming led to the community's divergent succession and accelerated temporal scaling^7^, ^8^, as well as reported in a montane community and a coastal ecosystem^42^, ^43^. Based on the metabolic theory of ecology, temperature determines metabolic rates and thus affects nearly all biological processes, including species turnover^44^, ^45^. Thus, the trend of an increase in community variation among months and across experimental plots can be directly predicted by elevated temperature. Such changes are potentially important for the seasonal dynamics of C fluxes and the overall C balance between land and atmosphere in the long term and should be taken into account by terrestrial ecosystem models.

In summary, this study revealed that climate warming altered the seasonal succession of microbial community structure and network patterns in a temperate tall‐grass prairie ecosystem. Although strong seasonal changes in soil microclimate and resource availability led to vastly different microbial network sizes and complexity across seasons, warming increased the complexity of the networks, with the most profound influence in colder months. The stronger warming effect on community structure and network characteristics in colder months were likely related to stronger deterministic selection and less significant stochasticity in community assembly imposed by soil moisture loss under warming. Furthermore, elevated temperature directly led to increased seasonal and spatial variation in soil microbial communities as predicted by the metabolic theory of ecology. This study demonstrated that the seasonal dynamics of the microbial community can be greatly restructured by warming. Such seasonality of microbial responses to warming may enlarge, potentially resulting in significant impacts on the terrestrial carbon cycle.

## MATERIALS AND METHODS

### Study site and experimental design

The field experiment is a part of the Kessler Atmospheric and Ecological Field Station (KAEFS), located in a tall‐grass prairie ecosystem of the US Great Plains in Central Oklahoma, USA (34̊°59ʹN, 97̊°31ʹW). As described previously^46^, ^47^, the experiment is in a temperate climate region with four distinct seasons and two plant biomass peaks. Spring is characterized by increasing temperature and precipitation, the germination and growth of C3 grasses, and forbs toward an earlier biomass peak, and usually spans mid‐February, March, and April till early or mid‐May. Summer is warm with the scenes of earlier vegetation cover and rapid growth of tall grasses, including *Ambrosia trifida* and *Solanum carolinense* belonging to C3 forbs, and *Tridens flavus*, *Sporobolus compositus*, and *Sorghum halapense* belonging to C4 grasses. It lasts from May to mid‐ or late‐August. In fall, the biomass of plant community reaches its second peak in September and gradually turns into litter in October, and the temperature dramatically drops in November. The winter months of December, January, and February are cold and windy, with residual litter covering the ground. The local mean annual temperature (1948−2012) was 16.3°C, with monthly means ranging from 3.5°C in January to 28.1°C in July. Annual precipitation was 895 mm, with monthly totals from 33 mm in January to 126 mm in May (based on Oklahoma Climatological Survey). Since July 2009, the temperature of experimental plots has been manipulated to simulate climate warming. Each of the four biological replicate blocks contains a pair of 2.5 × 1.75 m plots, one for experimental warming treatment and the other for control. One infrared heater (165 × 9 × 15 cm; Kalglo Electronics) was installed 1.5 m above each warmed plot to achieve warming of the whole ecosystem. In each control plot, a woody bar of the same dimensions was installed as a “dummy” heater to mimic the shading effect.

### Field measurements, soil sampling, and physical‐chemical analysis

Soil temperature at 7.5 cm was recorded every 15 min by thermocouples (T‐type; Campbell Science Inst.) installed at the center of each plot^7^. Soil volumetric water content was measured at a depth of 0−15 cm every 30 min by TDR meters (ESI Environmental Sensors Inc.) installed in each plot. In accordance with the monthly soil sampling, monthly averages of soil temperature and moisture were calculated and further analyzed in this study. As described previously^46^, ^47^, soil *R*_t_ and its *R*_h_ and *R*_a_ were measured monthly. An LI‐6400 Portable Photosynthesis System (LI‐COR. Inc.) was used to measure monthly ecosystem C exchanges (e.g., NEE, *R*_eco_, and GPP)^47^.

One surface (0−15 cm) soil sample core (2.5 cm diameter) was taken monthly from each of the four warmed plots and four control plots for soil physical‐chemical and microbial analysis in 2012. Therefore, a total of 96 monthly soil samples were collected in this study and stored in −80°C freezers after each sampling event until processing. The samples from different months were grouped as follows based on the local weather condition: spring from March to May, summer from June to August, fall from September to November, and winter covering January, February, and December. Thus, there are 12 warmed samples and 12 control samples in each season.

Visible roots and stones were picked out from soil samples before processing. Soil pH was measured at a 1:5 soil‐to‐water mass ratio mixture using an Accumet Excel XL15 pH Meter (Fisher Scientific) with a combined calibrated electrode. For determining SOC and TN, the dried samples were treated with 1 N HCl for 24 h to remove soil inorganic C (carbonates) and applied to a dry combustion C and N analyzer (LECO). Soil NH_4_^+^ and NO_3_^−^ concentrations were measured by a Lachat 8000 flow‐injection analyzer (Lachat).

### DNA extraction, PCR, and Illumina sequencing

Soil total DNA was extracted by a freeze‐grinding method followed by the SDS‐based lysis and phenol‐chloroform extraction^48^, and purified through the columns provided in the MoBio PowerSoil DNA isolation kit (MoBio Laboratories). The quality and purity of DNA was assessed by a NanoDrop ND‐1000 Spectrophotometer (NanoDrop Technologies Inc.). The final DNA concentrations were quantified by PicoGreen using a FLUOstar OPTIMA fluorescence plate reader (BMG LabTech).

To determine the microbial composition, a 16S riobosomal RNA (rRNA) gene amplicon library was prepared and sequenced by an Illumina MiSeq platform^49^. DNA was amplified by PCR in triplicates for the V4 region of 16S rRNA genes using the Illumina adapted primer set 515F and 806R according to a previous protocol^50^. The amplicons from each sample were gel‐purified and further used to prepare the sequencing library. The library was then sequenced on an Illumina MiSeq.

Data processing of the 16S rRNA gene was performed in the pipeline developed by the Institute for Environmental Genomics (IEG) (http://zhoulab5.rccc.ou.edu:8080). More than 8 million raw sequences were retrieved by matching the sample barcodes, and primer sequences at the end of each read were trimmed. OTUs were clustered by UPARSE^51^ at 97% identity, and singletons were removed from the remaining sequences. In UPARSE, the green reference data set^52^ for the 16S rRNA gene was used as a reference database to remove chimeras. Finally, the OTU table was randomly subsampled so that the total sequence number in each sample was 17,700. A total of 18,333 OTUs were obtained from all 96 samples. The OTU taxonomic classification was performed using representative sequences from each OTU through the Ribosomal Database Project (RDP) Classifier with 50% confidence estimates^53^.

### Microbial ecological network analysis

Microbial ecological networks were constructed by using the Molecular Ecological Network Analyses pipeline (MENAP, http://ieg2.ou.edu/MENA/)^54^. First, two global networks, one for control treatment, and the other one for warming treatment were constructed, each containing 48 samples from all field replicates across all months. The OTUs present in 36 or more (≥75%) samples were included in each network construction to obtain robust data association estimations. Then, eight seasonal networks were constructed, each containing 12 samples from four field replicates across 3 months of each season. For the seasonal networks, the OTUs occurring in at least nine samples (≥75%) were used for construction.

The network topological properties, module separation, key taxa identification, and eigengene analysis were all performed in the MENA pipeline as previously described^54^. Networks were visualized using Gephi 0.9.1 and Cytoscape 3.5.0. The nodes in each network were separated into modules using the Greedy modularity optimization algorithm^55^. A module is a group of nodes (e.g., OTUs) that are highly connected within the group but have few connections with nodes outside the group. Modularity (*M*) > 0.4 was used as the threshold to define modular structures^55^. For each node, its within‐module connectivity (*Z*_*i*_) and among module connectivity (*P_i_*) were calculated^56^, which were used to identify module hubs, network hubs, and connectors as key species that played important roles in the network topological structure. Module hubs are the highly connected nodes within modules (*Z*_*i*_ > 2.5), network hubs are highly connected nodes within the entire network (*Z*_*i*_ > 2.5, *P_i_* > 0.62), and connectors are nodes that connect modules (*P_i_* > 0.62). In addition to these three categories, the other nodes belong to peripherals that are connected in modules with few outside connections (*Z*_*i*_ < 2.5, *P_i_* < 0.62).

To reveal higher‐order organization among modules in a network, eigengene analysis was performed by using the large modules containing ≥5 nodes. An eigengene was calculated based on methods as previously described^54^ to summarize the species abundance information from a module as a centroid. All eigengenes from the same network, each representing one major module in the network, were then used for the hierarchical clustering analysis to assess the organization or relationship of different modules in one network. Furthermore, the correlation of each eigengene−environmental variable pair was calculated as Pearson's product−moment to assess the potential associations of each module to the environments.

To evaluate the robustness of the microbial network, simulations of random or targeted species removal were performed in this study. A certain proportion of nodes was randomly removed to simulate random species removal. Meanwhile, certain numbers of module hubs were removed to simulate targeted removal. To test the effects of species removal on the remaining species of the network, the abundance‐weighted mean interaction strength of the node was calculated as previously described^9^. After removing the selected species in the network, the proportion of the remaining nodes was defined as the network robustness. We reported the robustness of the network when 50% of random nodes or five module hubs were removed in this study.

### Community assembly mechanisms

To disentangle the importance of deterministic mechanisms from stochastic mechanisms underlying microbial community assembly, NST was calculated based on taxonomic metrics using the R package NST^57^. Since the taxonomic metrics are not independent (pairwise comparisons), permutational multivariate analysis of variance (PERMANOVA) considering the repeated‐measures design (1000 permutations) was performed to evaluate the significance of treatment and month in this study.

### Statistical analysis

To test the effects of experimental warming and time on environmental variables, microbial communities, and their network properties, as well as to assess the correlations among the three aspects, the following analyses were performed: (1) Microbial β‐diversity was assessed by using the Bray−Curtis distance metrics, and the distances of paired warmed and control plots within each block over time were fitted to a nonlinear quadratic regression. (2) PCoA of soil bacterial community structures was performed to visualize microbial community patterns under warming in different seasons. (3) A multivariate permutational procedure, the PERMANOVA^58^, was used to test how different the entire microbial community structures were under different treatment and sampling time. Bray−Curtis distance was used to calculate the distance matrix in this test. (4) The test of multivariate homogeneity of group variances^59^ was used to calculate the Sørensen distances of microbial communities to their group centroids based on treatment, sampling month, as well as sampling season. The difference in the average distances to group centroids indicates beta‐dispersion or heterogeneity of communities among groups. This analysis was also used to infer variation of soil variables and C fluxes within the control or warmed plots. (5) CCA and Mantel tests were used to link the microbial community structures to soil variables and ecosystem functions. (6) Linear mixed‐effects models with block as a random intercept effect were used to analyze the effects of warming and sampling months on the following variables: soil variables, soil respirations and ecosystem fluxes, microbial α‐diversity indexes, and the abundances of different microbial phyla. The effects of warming on the topological properties of networks were also analyzed using linear mixed‐effects models. All the statistical analyses were performed using R software 3.5.1 with packages vegan, picante, nlme, and lmerTest.

## AUTHOR CONTRIBUTIONS

All authors contributed intellectual input and assistance to this study and manuscript preparation. Research questions and experimental strategy were developed by Jizhong Zhou. Field management was carried out by Liyou Wu, Mengting Yuan, Xue Guo, Xishu Zhou, and Jiabao Li. Sample collection, DNA preparation, and MiSeq sequencing analysis were carried out by Mengting Yuan, Zhou Shi, Xishu Zhou, and Jiabao Li. Soil chemical analyses were carried out by Mengting Yuan, Xishu Zhou, Jiabao Li, and Liyou Wu. Various statistical analyses were carried out by Xue Guo, Jiesi Lei, Mengting Yuan, and Ye Deng. Assistance in data interpretation was provided by Yunfeng Yang. All data analyses and integration were guided by Jizhong Zhou. The paper was written by Xue Guo and Mengting Yuan with the help of Jizhong Zhou and Yunfeng Yang. Considering their contributions in terms of data collection, analyses, and/or integration, Xue Guo, Mengting Yuan, and Jiabao Lei are listed as co‐first authors.

## ETHICS STATEMENT

This article does not contain any studies with human participants or animals performed by any of the authors.

## CONFLICT OF INTERESTS

The authors declare no conflict of interests.

## Supporting information

Supporting information.

## Data Availability

DNA sequences of 16S rRNA gene amplicons were available in NCBI Sequence Read Archive under project no. PRJNA626428. All other relevant data are available in Supporting Information.
