# Supplementary material for: Climate warming restructures seasonal dynamics of grassland soil microbial communities
Source: mLife. 2022 Sep 15;1(3):245–56. doi: 10.1002/mlf2.12035 (PMC10989843; doi:10.1002/mlf2.12035)
Supplement: Supplementary file 1 — Supporting information. [file MLF2-1-245-s001.pdf]

Supplementary Figures and tables

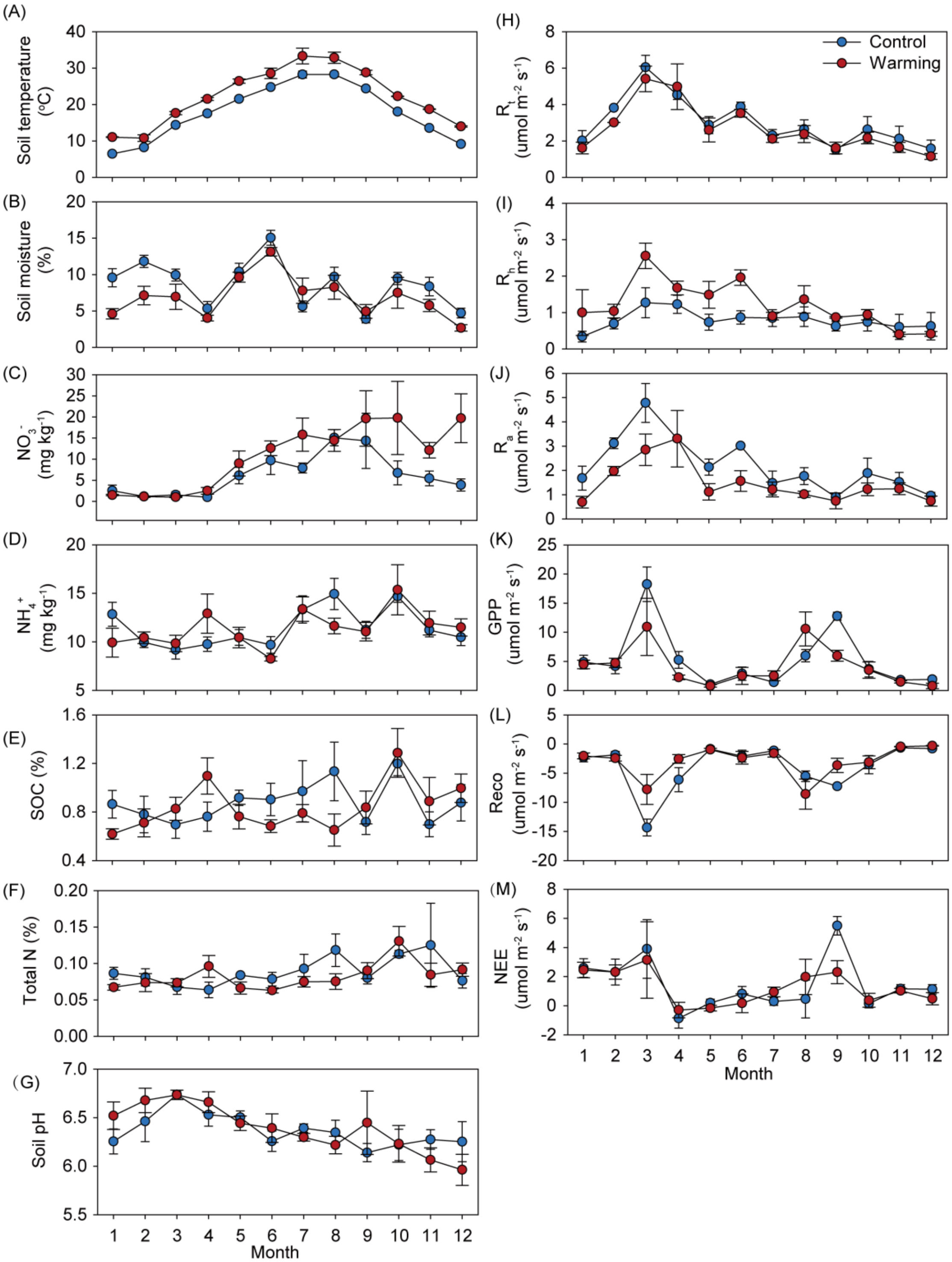

Figure S1. Monthly changes of soil variables (A-G), soil respirations (H-J) and ecosystem carbon fluxes (K-M) under warming and control. Dots represent the averaged values for measurements in each calendar month from 4 replicated plots and error bars are standard errors. The detailed information of these measurements is provided in Figure 1. The significances of warming treatment, sampling month, or their interaction are summarized in Table S1.

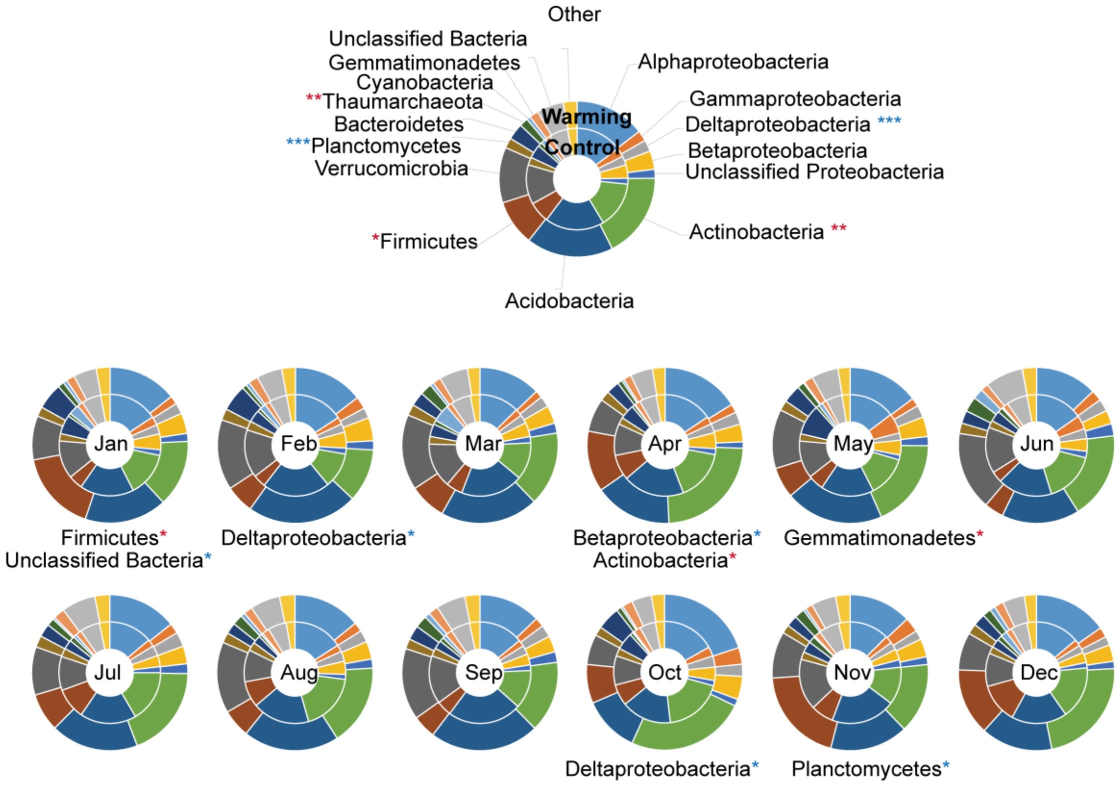

Figure S2. Microbial taxonomic compositions at phylum level under warming (outer circle) and control (inner circle) treatments across all months or from each month. Pie fractions indicate the relative abundances of phyla (classes for Proteobacteria) in terms of retrieved sequences numbers. Phyla shown below the pie chart of each month are those with a significant increase or decrease of relative abundance under warming. Read asterisks mark phyla with a significant increase of relative abundance under warming, while blue asterisks mark phyla with a significant decrease of relative abundance. The differences between warming and control are tested by linear mixed-effects models, indicated by *** when $p < 0.001$, ** when $p < 0.01$, * when $p < 0.05$.

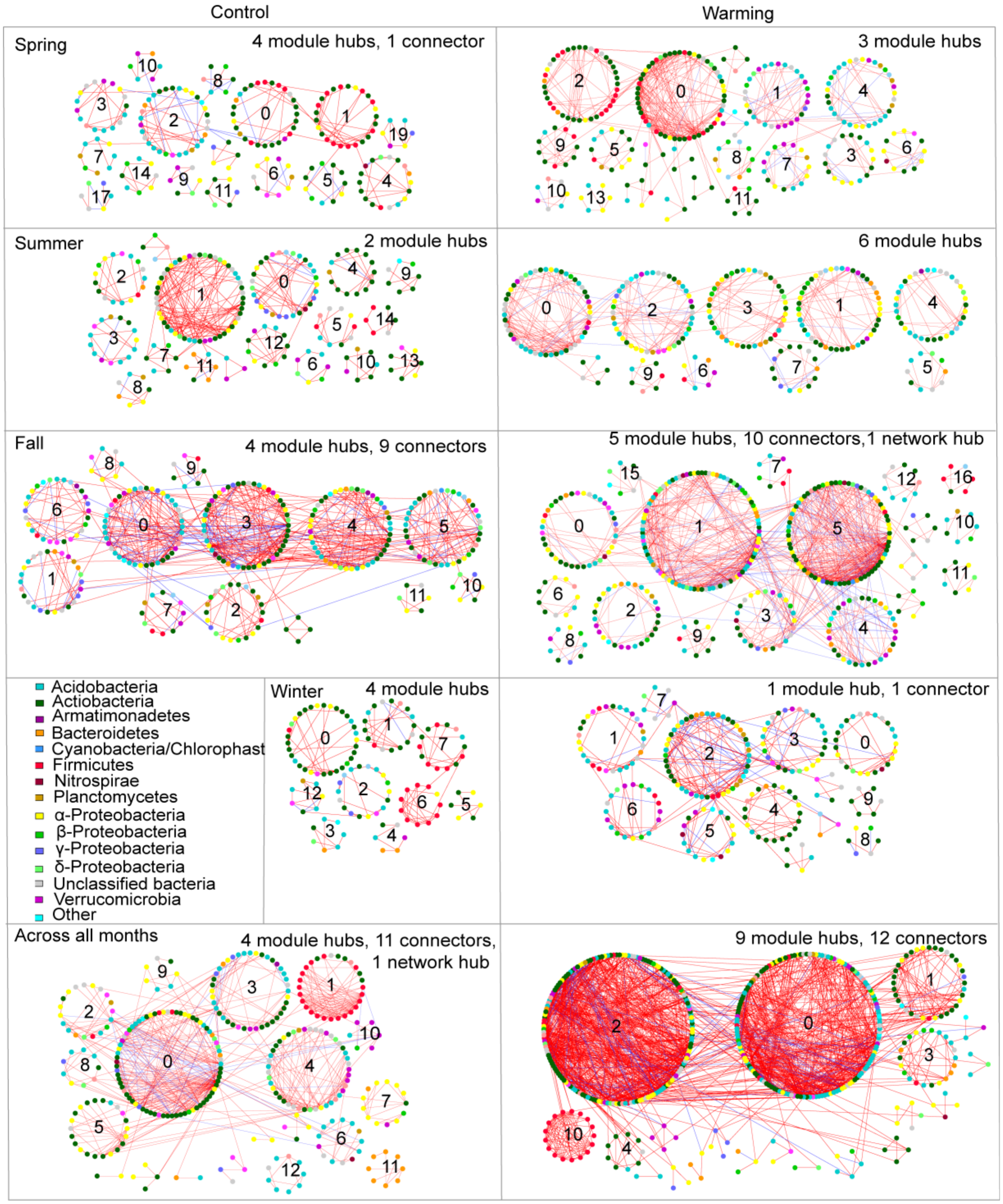

Figure S3. Highly connected modules from seasonal and global networks under warming and control treatments. In each network, modules with ⩾5 nodes are represented by circularly arranged nodes. Small modules with <5 nodes are only shown if they are linked to larger modules. Nodes are colored based on their taxa. Positive links are in red and negative links are in blue. An ID was assigned to each module and was shown in the center of each module.

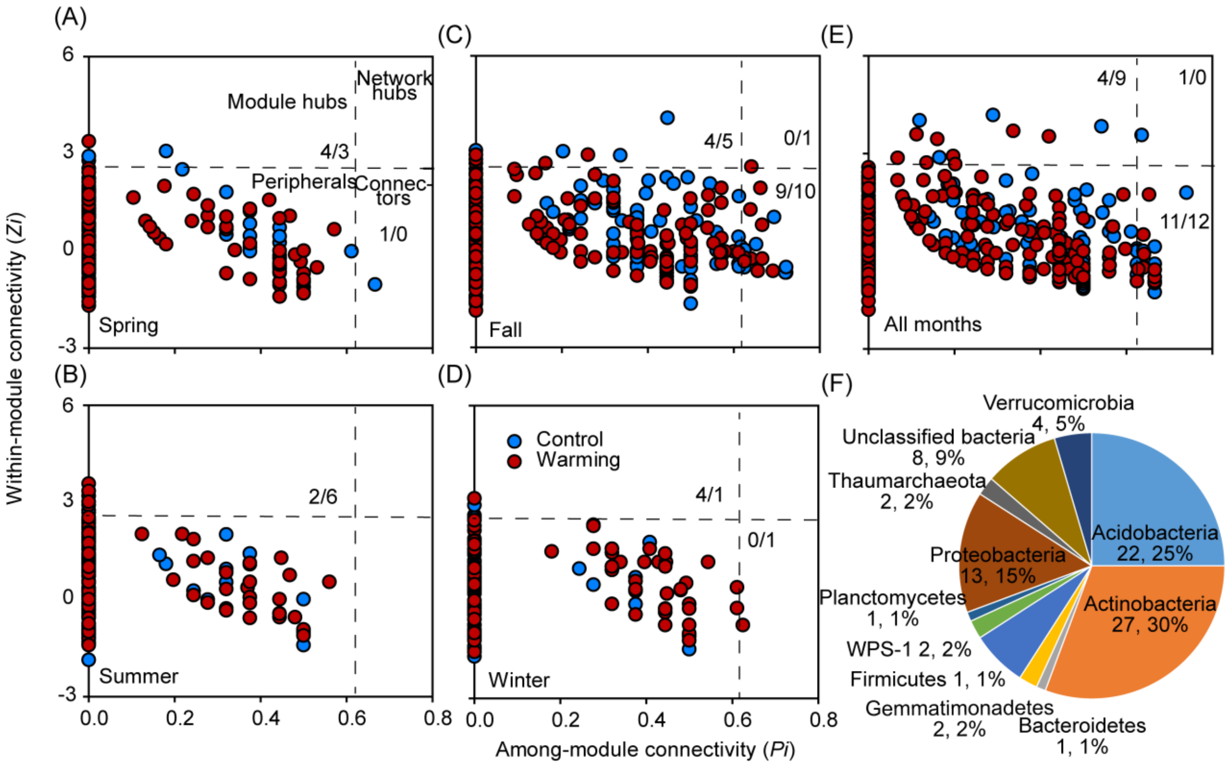

Figure S4. Key taxa of networks in different seasons and across all months. (**A-E**) *Zi-Pi* plots to identify putative keystone taxa under warming (red) and control (blue) treatments. The numbers of module hubs, connectors and the network hub in control/warmed networks are marked in corresponding quadrants separated by *Zi*=2.5 and *Pi*=0.62. (**F**) The phylogenetic profile of keystone OTUs identified in all networks. Numbers below phyla names are the number of OTUs and percentage in all module hubs, connectors and network hubs. Their detailed taxonomic information is listed in Table S3.

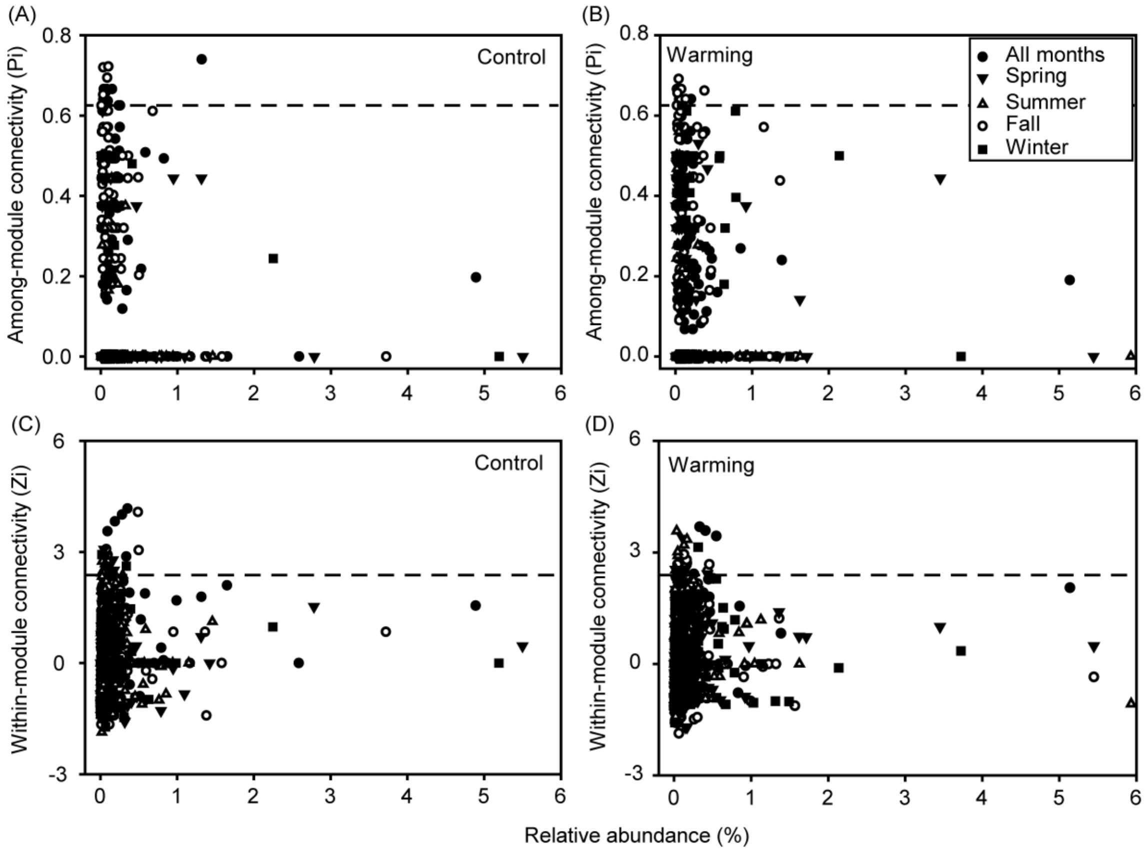

Figure S5. The relative abundances of module hubs and connectors in all networks under warming and control treatments. The dotted line indicated *Pi* = 0.62 in the above two plots to identify putative connectors under control (**A**) and warming (**B**) treatments, and the dotted line indicated *Zi* = 2.5 in the below two plots to identify putative module hubs under control (**C**) and warming (**D**) treatments.

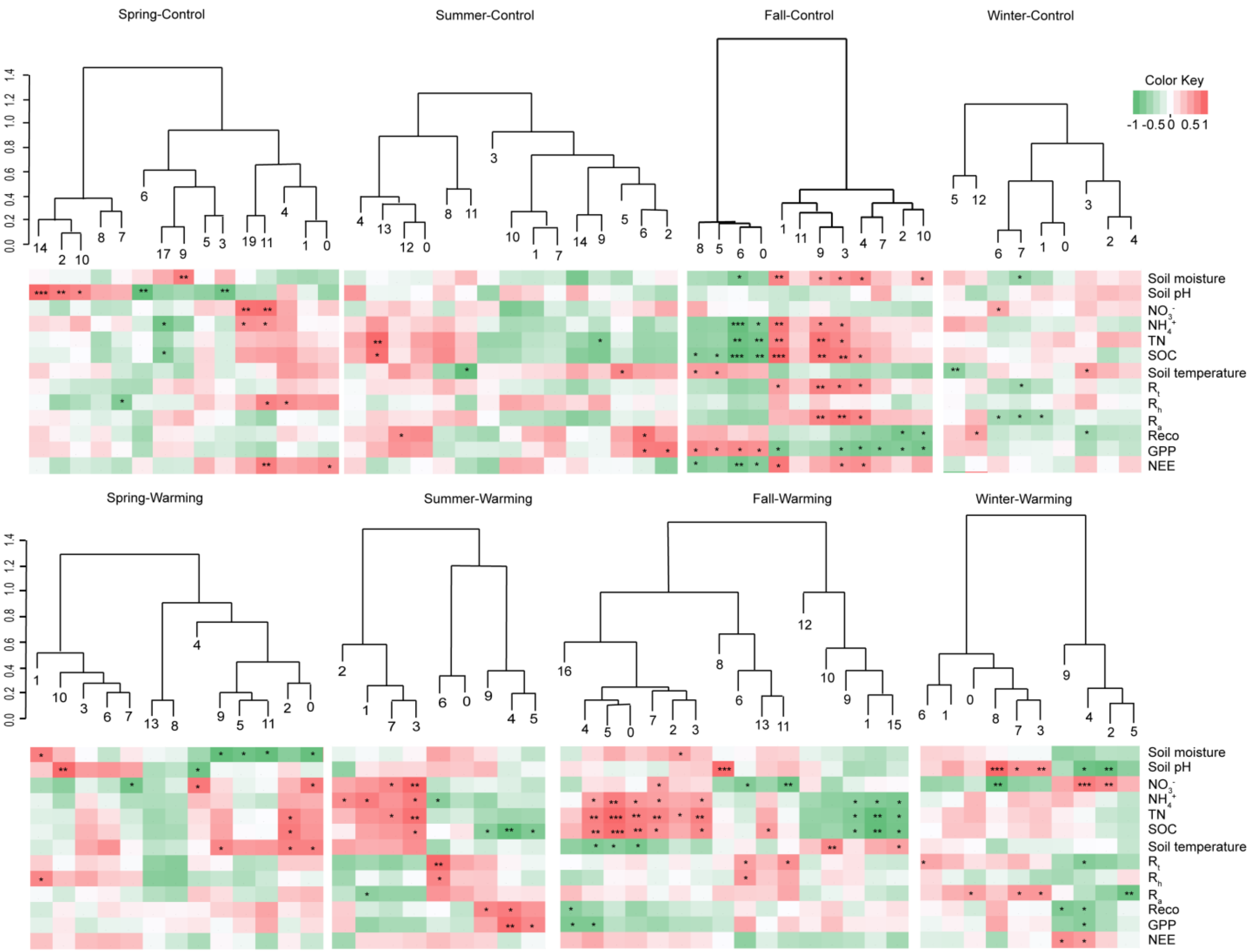

Figure S6. The correlations between module eigengenes with soil variables, soil respirations and ecosystem C fluxes in the seasonal networks. An eigengene was calculated for each module to represent all nodes within each module. Clusters showed the hierarchical clustering of eigengenes for modules numbered as in Figure S3. The Pearson correlation coefficient of each eigengene-variable pair was indicated by the color key. Significances of these correlations were indicated by *** when $p < 0.001$, ** when $p < 0.01$, * when $p < 0.05$. Soil variables, soil respirations and ecosystem C fluxes were defined as in the legend of Figure 1.

Table S1. The effects of warming and months on soil variables, soil respirations and ecosystem C fluxes based on linear mixed-effects models.

	Warming		Month		Warming:Month	
	F	*p*	F	*p*	F	*p*
Soil temperature	**218.109**	**<0.001**	**241.204**	**<0.001**	0.602	0.821
Soil moisture	**15.939**	**<0.001**	**13.568**	**<0.001**	1.735	0.084
Soil pH	0.169	0.682	**3.718**	**<0.001**	0.970	0.482
NO_3^-	**11.719**	**0.001**	**6.329**	**<0.001**	1.478	0.160
NH_4^+	0.042	0.838	**4.770**	**<0.001**	1.245	0.275
TN	1.035	0.313	1.804	0.070	1.060	0.406
SOC	0.335	0.565	**2.248**	**0.021**	1.578	0.125
R_t	3.269	0.075	**19.563**	**<0.001**	0.293	0.985
R_h	**21.103**	**<0.001**	**6.771**	**<0.001**	**2.079**	**0.033**
R_a	**23.624**	**<0.001**	**13.118**	**<0.001**	1.254	0.270
Reco	3.675	0.059	**15.465**	**<0.001**	**2.489**	**0.011**
GPP	3.166	0.080	**13.689**	**<0.001**	**2.233**	**0.022**
NEE	0.438	0.511	**4.977**	**<0.001**	0.806	0.634

Significances (p <0.05) are shown in bold. The details of these variables were provided in Figure 1.

Table S2. Topological properties of soil microbial co-occurrence networks under warming (before slash) and control (after slash) in separate seasons and across all months.

Sampling time		Spring	Summer	Fall	Winter	Across months
	Numbers of OTUs*	1240/1312	1276/1356	1176/1419	1155/1342	1056/1180
Empirical network	Similarity threshold	0.89/0.89	0.89/0.89	0.89/0.89	0.89/0.89	0.68/0.68
	Total nodes	489/426	456/450	569/528	408/394	453/349
	Total links	668/377	567/464	1096/746	451/311	1678/693
	R square of power law	0.918/0.921	0.949/0.961	0.937/0.948	0.935/0.977	0.850/0.927
	Average Connectivity (avgK)	2.732/1.770	2.487/2.062	3.852/2.826	2.211/1.579	7.408/3.971
	Average clustering coefficient (avgCC)	0.159/0.106	0.153/0.139	0.195/0.173	0.150/0.092	0.345/0.260
	Average path distance (GD)	9.531/8.987	8.402/3.572	5.540/5.376	6.769/7.681	4.394/5.038
	Harmonic geodesic distance (HD)	6.103/5.760	5.937/2.617	4.299/4.393	5.136/3.971	3.751/4.167
	Geodesic efficiency (E)	0.164/0.174	0.168/0.382	0.233/0.228	0.195/0.252	0.267/0.240
	Connectance (Con)	0.293/0.135	0.307/0.039	0.359/0.299	0.261/0.043	0.926/0.715
	Centralization of degree (CD)	0.040/0.019	0.036/0.029	0.053/0.044	0.027/0.014	0.066/0.095
	No. of modules	114/117	91/117	96/110	92/128	28/37
	Modularity (M)	0.789/0.914	0.840/0.877	0.673/0.754	0.814/0.945	0.522/0.680
	Relative modularity (RM)	0.208/0.030	0.188/0.094	0.330/0.165	0.067/0.019	0.642/0.379
	Positive links	619/335	523/449	927/628	375/283	1545/646
	Negative links	49/42	44/15	169/118	76/28	133/47
	Proportion (Positive/total)	0.927/0.889	0.922/0.967	0.846/0.842	0.831/0.910	0.921/0.932
Random networks**	avgCC ± SD	0.011±0.003/ 0.002±0.002	0.007±0.003/ 0.005±0.003	0.021±0.004/ 0.009±0.003	0.005±0.003/ 0.003±0.002	0.046±0.004/ 0.032±0.006
	GD ± SD	4.679±0.077/ 8.444±0.631	5255±0.104/ 5.973±0.204	4.052±0.050/ 4.875±0.077	5.740±0.166/ 8.277±1.118	3.248±0.022/ 3.819±0.048
	E ± SD	0.239±0.003/ 0.152±0.009	0.216±0.004/ 0.196±0.005	0.271±0.002/ 0.229±0.003	0.202±0.005/ 0.179±0.023	0.337±0.002/ 0.290±0.003
	HD ± SD	4.181±0.052/ 6.583±0.389	4.631±0.078/ 5.103±0.138	3.684±0.033/ 4.363±0.052	4.947±0.113/ 5.676±0.703	2.966±0.014/ 3.443±0.032
	Con ± SD	0.719±0.032/ 0.351±0.037	0.703±0.039/ 0.534±0.037	0.858±0.025/ 0.768±0.028	0.618±0.039/ 0.146±0.034	0.983±0.012/ 0.924±0.027
	M ± SD	0.653±0.006/ 0.887±0.008	0.707±0.006/ 0.802±0.007	0.506±0.005/ 0.647±0.006	0.763±0.008/ 0.927±0.007	0.318±0.004/ 0.493±0.006

*The majority rules of selecting OTUs for network construction are to include OTUs present in >75% (9) samples for separate seasons data sets, and those present in >75% (36) samples for combined-months data sets.

**100 random networks were generated by rewiring all the links of a corresponding empirical network with the identical numbers of nodes and links. The parameters presented here were the mean values and standard derivations from 100 random networks.

Table S3 Taxonomic information of module hubs, connectors and network hubs.

OTU	Network/module	Domain	Phylum	Class	Order	Family	Genus	Relative abundance (%)
**Module hubs**								
OTU_74	Spring_control/0	Bacteria	Actinobacteria	Actinobacteria	Actinomycetales	Streptomycetaceae	Streptomyces	0.164
OTU_22	Spring_control/1	Bacteria	Firmicutes	Bacilli	Bacillales	Unclassified	Unclassified	0.178
OTU_217	Spring_control/2	Bacteria	Acidobacteria	Acidobacteria Gp25	Unclassified	Unclassified	Gp25	0.048
[2]OTU_1982	Spring_control/4	Bacteria	Actinobacteria	Actinobacteria	Solirubrobacterales	Solirubrobacteraceae	Solirubrobacter	0.067
OTU_81	Spring_warming/0	Bacteria	Actinobacteria	Actinobacteria	Unclassified	Unclassified	Unclassified	0.148
OTU_586	Spring_warming/0	Bacteria	Firmicutes	Bacilli	Bacillales	Paenibacillaceae 1	Paenibacillus	0.019
[2]OTU_301	Spring_warming/4	Bacteria	Unclassified	Unclassified	Unclassified	Unclassified	Unclassified	0.114
OTU_567	Summer_control/1	Bacteria	Deltaproteobacteria	Deltaproteobacteria	Unclassified	Unclassified	Unclassified	0.024
OTU_342	Summer_control/4	Bacteria	Actinobacteria	Actinobacteria	Actinomycetales	Nocardioidaceae	Marmoricola	0.062
OTU_247	Summer_warming/0	Bacteria	Unclassified	Unclassified	Unclassified	Unclassified	Unclassified	0.128
OTU_564	Summer_warming/1	Bacteria	Actinobacteria	Actinobacteria	Actinomycetales	Micromonosporaceae	Catellatospora	0.033
OTU_476	Summer_warming/2	Bacteria	Acidobacteria	Acidobacteria Gp3	Unclassified	Unclassified	Gp3	0.048
OTU_51	Summer_warming/2	Bacteria	Gemmatimonadetes	Gemmatimonadetes	Gemmatimonadales	Gemmatimonadaceae	Gemmatimonas	0.166
OTU_6744	Summer_warming/3	Bacteria	Alphaproteobacteria	Alphaproteobacteria	Rhizobiales	Xanthobacteraceae	Pseudolabrys	0.047
OTU_376	Summer_warming/0	Bacteria	Unclassified	Unclassified	Unclassified	Unclassified	Unclassified	0.022
[3]OTU_15	Fall_control/0	Bacteria	Acidobacteria	Acidobacteria Gp1	Unclassified	Unclassified	Gp1	0.499
[3]OTU_21	Fall_control/3	Bacteria	Actinobacteria	Actinobacteria	Solirubrobacterales	Solirubrobacteraceae	Solirubrobacter	0.489
OTU_198	Fall_control/4	Bacteria	Actinobacteria	Actinobacteria	Actinomycetales	Micromonosporaceae	Unclassified	0.095
OTU_222	Fall_control/5	Bacteria	Deltaproteobacteria	Deltaproteobacteria	Myxococcales	Unclassified	Unclassified	0.077
[3]OTU_15	Fall_warming/1	Bacteria	Acidobacteria	Acidobacteria Gp1	Unclassified	Unclassified	Gp1	0.458
OTU_110	Fall_warming/1	Bacteria	Gemmatimonadetes	Gemmatimonadetes	Gemmatimonadales	Gemmatimonadaceae	Gemmatimonas	0.044
OTU_292	Fall_warming/5	Bacteria	Actinobacteria	Actinobacteria	Actinomycetales	Micromonosporaceae	Micromonospora	0.163
[2]OTU_299	Fall_warming/5	Bacteria	Actinobacteria	Actinobacteria	Solirubrobacterales	Conexibacteraceae	Conexibacter	0.114
OTU_260	Fall_warming/5	Bacteria	Actinobacteria	Actinobacteria	Actinomycetales	Pseudonocardiaceae	Actinomycetospora	0.129
[3]OTU_21	Winter_control/0	Bacteria	Actinobacteria	Actinobacteria	Solirubrobacterales	Solirubrobacteraceae	Solirubrobacter	0.339
[2]OTU_1982	Winter_control/0	Bacteria	Actinobacteria	Actinobacteria	Solirubrobacterales	Solirubrobacteraceae	Solirubrobacter	0.096
OTU_14931	Winter_control/1	Bacteria	Firmicutes	Bacilli	Bacillales	Paenibacillaceae 1	Cohnella	0.020
OTU_2006	Winter_control/7	Bacteria	Firmicutes	Bacilli	Bacillales	Bacillaceae 1	Bacillus	0.109
[2]OTU_30	Winter_warming/6	Bacteria	Acidobacteria	Acidobacteria Gp6	Unclassified	Unclassified	Gp6	0.311

[3]OTU_21	Global_control/0	Bacteria	Actinobacteria	Actinobacteria	Solirubrobacterales	Solirubrobacteraceae	Solirubrobacter	0.353
OTU_176	Global_control/0	Bacteria	Actinobacteria	Actinobacteria	Solirubrobacterales	Solirubrobacteraceae	Solirubrobacter	0.282
OTU_160	Global_control/3	Bacteria	Acidobacteria	Acidobacteria Gp1	Unclassified	Unclassified	Gp1	0.190
OTU_11	Global_control/5	Bacteria	Alphaproteobacteria	Alphaproteobacteria	Rhizobiales	Rhodobiaceae	Unclassified	0.337
[3]OTU_15	Global_warming/0	Bacteria	Acidobacteria	Acidobacteria Gp1	Unclassified	Unclassified	Gp1	0.548
OTU_73	Global_warming/0	Bacteria	Actinobacteria	Actinobacteria	Gaiellales	Gaiellaceae	Gaiella	0.167
OTU_80	Global_warming/0	Bacteria	Acidobacteria	Acidobacteria Gp1	Unclassified	Unclassified	Gp1	0.087
OTU_27	Global_warming/0	Bacteria	Acidobacteria	Acidobacteria Gp1	Unclassified	Unclassified	Gp1	0.406
[2]OTU_301	Global_warming/0	Bacteria	Unclassified	Unclassified	Unclassified	Unclassified	Unclassified	0.122
OTU_40	Global_warming/1	Bacteria	Actinobacteria	Actinobacteria	Gaiellales	Gaiellaceae	Gaiella	0.331
OTU_221	Global_warming/2	Bacteria	Unclassified	Unclassified	Unclassified	Unclassified	Unclassified	0.068
OTU_17316	Global_warming/2	Bacteria	Acidobacteria	Acidobacteria Gp4	Unclassified	Unclassified	Gp4	0.099
OTU_206	Global_warming/3	Bacteria	Bacteroidetes	Sphingobacteriia	Sphingobacteriales	Chitinophagaceae	Flavitalea	0.073
**Connectors**								
[3]OTU_125	Spring_control/2	Bacteria	WPS-1	Unclassified	Unclassified	Unclassified	WPS-1 genera	0.110
OTU_63	Fall_control/0	Bacteria	Alphaproteobacteria	Alphaproteobacteria	Rhodospirillales	Unclassified	Unclassified	0.100
OTU_396	Fall_control/0	Bacteria	Actinobacteria	Actinobacteria	Actinomycetales	Mycobacteriaceae	Mycobacterium	0.034
OTU_349	Fall_control/1	Bacteria	Verrucomicrobia	Subdivision3	Unclassified	Unclassified	Subdivision3	0.028
OTU_1383	Fall_control/1	Bacteria	Planctomycetes	Planctomycetia	Planctomycetales	Planctomycetaceae	Unclassified	0.016
OTU_978	Fall_control/2	Bacteria	Actinobacteria	Actinobacteria	Gaiellales	Gaiellaceae	Gaiella	0.012
OTU_940	Fall_control/4	Bacteria	Acidobacteria	Acidobacteria Gp6	Unclassified	Unclassified	Gp6	0.031
[2]OTU_299	Fall_control/5	Bacteria	Actinobacteria	Actinobacteria	Solirubrobacterales	Conexibacteraceae	Conexibacter	0.086
OTU_15079	Fall_control/5	Bacteria	Actinobacteria	Actinobacteria	Actinomycetales	Streptomycetaceae	Streptacidiphilus	0.099
OTU_1169	Fall_control/8	Bacteria	Alphaproteobacteria	Alphaproteobacteria	Rhizobiales	Roseiarcaceae	Roseiarcus	0.009
OTU_15155	Fall_warming/0	Archaea	Thaumarchaeota	Unclassified	Nitrososphaerales	Nitrososphaeraceae	Nitrososphaera	0.048
OTU_11606	Fall_warming/0	Bacteria	Acidobacteria	Acidobacteria Gp5	Unclassified	Unclassified	Gp5	0.052
OTU_654	Fall_warming/2	Bacteria	Actinobacteria	Actinobacteria	Acidimicrobiales	Iamiaceae	Aquihabitans	0.047
OTU_264	Fall_warming/3	Bacteria	Alphaproteobacteria	Alphaproteobacteria	Rhizobiales	Methylobacteriaceae	Microvirga	0.069
OTU_1	Fall_warming/4	Bacteria	Verrucomicrobia	Spartobacteria	Unclassified	Unclassified	Spartobacteria	5.449
[2]OTU_30	Fall_warming/4	Bacteria	Acidobacteria	Acidobacteria Gp6	Unclassified	Unclassified	Gp6	0.379
[2]OTU_9432	Fall_warming/4	Bacteria	Acidobacteria	Acidobacteria Gp6	Unclassified	Unclassified	Gp6	0.078
OTU_15212	Fall_warming/4	Bacteria	Unclassified	Unclassified	Unclassified	Unclassified	Unclassified	0.022
OTU_399	Fall_warming/5	Bacteria	Actinobacteria	Actinobacteria	Actinomycetales	Microbacteriaceae	Lysinimonas	0.043
OTU_1597	Fall_warming/5	Bacteria	Actinobacteria	Actinobacteria	Actinomycetales	Micrococcaceae	Unclassified	0.028

[3]OTU_125	Winter_warming/2	Bacteria	WPS-1	Unclassified	Unclassified	Unclassified	WPS-1 genera	0.079
OTU_224	Global_control/0	Bacteria	Betaproteobacteria	Betaproteobacteria	Unclassified	Unclassified	Unclassified	0.058
OTU_255	Global_control/2	Bacteria	Acidobacteria	Acidobacteria Gp5	Unclassified	Unclassified	Gp5	0.033
OTU_195	Global_control/3	Bacteria	Verrucomicrobia	Subdivision3	Unclassified	Unclassified	Subdivision3	0.235
OTU_150	Global_control/3	Bacteria	Gammaproteobacteria	Gammaproteobacteria	Xanthomonadales	Sinobacteraceae	Povalibacter	0.079
[2]OTU_9432	Global_control/3	Bacteria	Acidobacteria	Acidobacteria Gp6	Unclassified	Unclassified	Gp6	0.149
OTU_560	Global_control/4	Bacteria	Actinobacteria	Actinobacteria	Unclassified	Unclassified	Unclassified	0.015
OTU_258	Global_control/6	Bacteria	Acidobacteria	Acidobacteria Gp5	Unclassified	Unclassified	Gp5	0.039
[2]OTU_1741	Global_control/8	Bacteria	Gammaproteobacteria	Gammaproteobacteria	Unclassified	Unclassified	Unclassified	0.040
OTU_33	Global_control/9	Bacteria	Alphaproteobacteria	Alphaproteobacteria	Alphaproteobacteria	Unclassified	Rhizomicrobium	0.254
[2]OTU_219	Global_control/9	Bacteria	Acidobacteria	Acidobacteria Gp6	Unclassified	Unclassified	Gp6	0.040
OTU_6	Global_control/10	Bacteria	Verrucomicrobia	Spartobacteria	Unclassified	Unclassified	Spartobacteria	1.316
OTU_121	Global_warming/0	Bacteria	Unclassified	Unclassified	Unclassified	Unclassified	Unclassified	0.069
OTU_23	Global_warming/1	Bacteria	Firmicutes	Unclassified	Unclassified	Unclassified	Unclassified	0.206
OTU_557	Global_warming/1	Bacteria	Actinobacteria	Unclassified	Unclassified	Unclassified	Unclassified	0.048
OTU_791	Global_warming/1	Bacteria	Actinobacteria	Actinobacteria	Actinomycetales	Nocardioidaceae	Unclassified	0.019
[2]OTU_219	Global_warming/1	Bacteria	Acidobacteria	Acidobacteria Gp6	Unclassified	Unclassified	Gp6	0.022
OTU_316	Global_warming/1	Bacteria	Unclassified	Unclassified	Unclassified	Unclassified	Unclassified	0.020
OTU_3392	Global_warming/2	Bacteria	Firmicutes	Unclassified	Unclassified	Unclassified	Unclassified	0.075
OTU_823	Global_warming/2	Bacteria	Actinobacteria	Actinobacteria	Actinomycetales	Nocardioidaceae	Nocardioides	0.030
OTU_13838	Global_warming/4	Bacteria	Acidobacteria	Acidobacteria Gp6	Unclassified	Unclassified	Gp6	0.055
OTU_34	Global_warming/5	Bacteria	Acidobacteria	Acidobacteria Gp1	Unclassified	Unclassified	Gp1	0.216
OTU_29	Global_warming/7	Bacteria	Gammaproteobacteria	Gammaproteobacteria	Xanthomonadales	Sinobacteraceae	Povalibacter	0.176
[2]OTU_1741	Global_warming/7	Bacteria	Gammaproteobacteria	Gammaproteobacteria	Unclassified	Unclassified	Unclassified	0.020
**Network hubs**								
OTU_105	Fall_warming/3	Archaea	Thaumarchaeota	Unclassified	Nitrososphaerales	Nitrososphaeraceae	Nitrososphaera	0.135
OTU_138	Global_control/2	Bacteria	Acidobacteria	Acidobacteria Gp5	Unclassified	Unclassified	Gp5	0.093

The superscript numbers before the OTU indicate the number of networks in which those OTUs are present as keystone taxa.

Table S4. The correlations of microbial community with soil variables, soil respirations and ecosystem C fluxes by Mantel test.

	Control		Warming	
Variables[+]	r	p	r	p
Soil temperature	-0.032	0.714	-0.011	0.554
Soil moisture	0.01	0.414	0.167	**0.008**
Soil pH	0.086	0.139	0.207	**0.003**
NO_3^-	-0.048	0.683	0.304	**0.007**
NH_4^+	0.067	0.199	0.299	**0.003**
TN	0.007	0.413	0.309	**0.002**
SOC	0.172	**0.044**	0.25	**0.003**
R_t	0.066	0.163	0.056	0.239
R_h	0.03	0.276	0.046	0.255
R_a	0.075	0.144	0.023	0.338
Reco	0.027	0.320	-0.095	0.855
GPP	0.031	0.283	-0.088	0.832
NEE	-0.072	0.769	-0.099	0.858

[+] Abbreviation of environmental attributes: SOC, soil organic carbon; TN, total nitrogen; R_t, soil total respiration; R_h, heterotrophic respiration; R_a, autotrophic respiration; GPP, gross primary productivity; Reco, ecosystem respiration; NEE, net ecosystem exchange.

Table S5. Summary of module eigengene analysis.

Network	Module numbers (≥5 nodes)[#]	Variation explained by eigengene (%)[#]
Spring-control	0/1/2/3/4/5/6/7/8/9/10/11/12/15/18/20	59/75/66/64/71/69/69/71/75/77/76/82/77/79/80 %
Summer-control	0/1/2/3/4/5/6/7/8/9/10/11/12/13/14	67/73/67/73/68/73/77/75/76/76/76/69/72/82/75 %
Fall-control	0/1/2/3/4/5/6/7/8/9/10/11	65/56/64/69/65/61/68/70/81/81/75/79 %
Winter-control	0/1/2/3/4/5/6/7/12	66/69/67/73/76/83/77/79/76 %
Spring-warming	0/1/2/3/4/5/6/7/8/9/10/11/13	71/69/65/66/65/68/71/72/67/79/65/63/80 %
Summer-warming	0/1/2/3/4/5/6/7/9	71/56/65/64/66/71/75/72/78 %
Fall-warming	0/1/2/3/4/5/6/7/8/9/10/11/12/13/15/16	63/64/63/62/64/72/77/79/79/83/78/71/81/77/82/81 %
Winter-warming	0/1/2/3/4/5/6/7/8/9	59/51/66/59/65/65/64/72/80/80 %
Global control	0/1/2/3/4/5/6/7/8/9/10/11/12	44/49/45/37/50/52/55/48/52/52/61/64/52 %
Global warming	0/1/2/3/4/9	34/42/39/46/64/69 %

[#] The module numbers and their variations explained by eigengene were separated by slash (/).
